# Three steps to writing adaptive study protocols in the early phase clinical development of new medicines

**DOI:** 10.1186/1471-2288-14-84

**Published:** 2014-06-30

**Authors:** Ulrike Lorch, Martin O’Kane, Jorg Taubel

**Affiliations:** 1 Richmond Pharmacology Ltd, St. George’s University of London, Cranmer Terrace, London, UK; 2 Medicines and Healthcare products Regulatory Agency, London, UK

**Keywords:** Adaptive study design, Adaptive protocol, Protocol writing, Early phase clinical research

## Abstract

This article attempts to define terminology and to describe a process for writing adaptive, early phase study protocols which are transparent, self-intuitive and uniform. It provides a step by step guide, giving templates from projects which received regulatory authorisation and were successfully performed in the UK. During adaptive studies evolving data is used to modify the trial design and conduct within the protocol-defined remit. Adaptations within that remit are documented using non-substantial protocol amendments which do not require regulatory or ethical review. This concept is efficient in gathering relevant data in exploratory early phase studies, ethical and time- and cost-effective.

## Findings

### Background

The use of adaptive study design in early exploratory clinical drug development, if thoroughly planned, is beneficial as it allows continuous learning from data that is being gathered. Thus, the study conduct can be adjusted accordingly within pre-specified boundaries, maximising the yield of useful information. Adaptations of the study conduct are protocol defined design features and not based on ad-hoc decisions [1]. An adaptive study protocol needs to be sufficiently detailed, clear and systematic whilst allowing for flexibility and evolution. Regulatory acceptability and efficient study conduct depend on a study protocol that is fit for purpose. It is desirable to define a uniform and intuitive terminology for adaptive protocols and to optimize a sufficiently comprehensive format, allowing the full assessment of risks and benefits of a proposed protocol, which can be easily followed in a global environment. The benefit of a standardised layout is that it facilitates ethical and regulatory review and makes subsequent adaptive protocol changes easy to document and follow.

In simple terms, there are three major elements to adaptive protocols in early phase drug development:

1. The description of the changes that can be made to study design and conduct, i.e. its *adaptive features*

2. The definition of the *boundaries* to these changes beyond which Regulatory and Ethics Committee approval needs to be obtained prior to implementation

3. The description of *control mechanisms* setting out how decisions will be made and how changes to the study will be managed and by whom

This article attempts to define terminology and to describe a clear process of writing an adaptive study protocol for the exploratory development of new medicines. It provides a step by step guide to protocol writing, including templates from projects we have authorised and performed in the UK. We have recently published an example which demonstrates the benefits of this concept [2]. Exploratory early phase trials are hypothesis forming, not hypothesis testing. Statistical analysis of these exploratory trials is descriptive in nature. Our paper does not aim to deal with statistical aspects of adaptive study design for confirmatory, hypothesis testing clinical trials. This manuscript describes a process and not research in human subjects, material or data, therefore it did not require REC approval.

### Regulatory background

There are few regulatory guidance documents on the topic, largely focused on later phase confirmatory studies. The European Medicines Agency (EMA) published a Reflection Paper on methodological issues in confirmatory clinical trials planned with an adaptive design (CHMP/EWP/2459/02) in 2007 [3]. The FDA published a draft Guidance for Industry: Adaptive Design Clinical Trials for Drugs and Biologics in February 2010 [4]. The FDA also published a draft Guidance for Industry: Enrichment Strategies for Clinical trials to support approval of human drugs and biological products in December 2012 which includes adaptive elements [5]. However, these guidance documents focus on confirmatory, hypothesis testing studies and do not address the specific issues surrounding adaptive design in exploratory early phase studies. There is paucity of publications describing the practical set-up and conduct of adaptive studies in early drug development.

## Discussion

### How to write an adaptive protocol

#### General process

Adaptive study design can be used in conventional early phase protocols comprising of just one element, such as a single ascending dose (SAD) protocol. Whilst the adaptive design principles can be used in any type of study, the full potential of adaptive study design can be exploited in combined or “umbrella” protocols. In an umbrella protocol a number of conventional studies (such as SAD, multiple ascending dose (MAD), food effect, drug-drug interaction, ethnic, age and/or gender comparison and cardiac safety studies etc.) are contained in one single study protocol.

The writing of an adaptive protocol commences with the description of the planned study design prior to any adaptations. At this stage the protocol looks similar to a non-adaptive study protocol. It will contain as a minimum a clear plan as to how to perform the dosing and assessments for the first subject(s) or the first dosing regimen. Equally, it may contain a plan for the entire study, including all anticipated dosing regimen and related assessments. After completing this initial “conventional” stage of protocol writing, the elements required by adaptive design are added, i.e. its *adaptive features, boundaries* and *control mechanisms.* They enable the study design to undergo pre-defined and justified evolutions so that for every study participant there is a valid and reproducible study plan.

### How to document adaptive changes to the protocol

All changes to the protocol, resulting from the implementation of pre-defined adaptive features, need to be fully documented.

Changes *within* the pre-defined scope, boundaries and control mechanisms of an adaptive study protocol can be documented as non-substantial protocol amendments or in administrative protocol change documents. In the UK these do not require notification to or authorisation by the Competent Authority (CA) or the Research Ethics Committee (REC).

**Figure 1:**
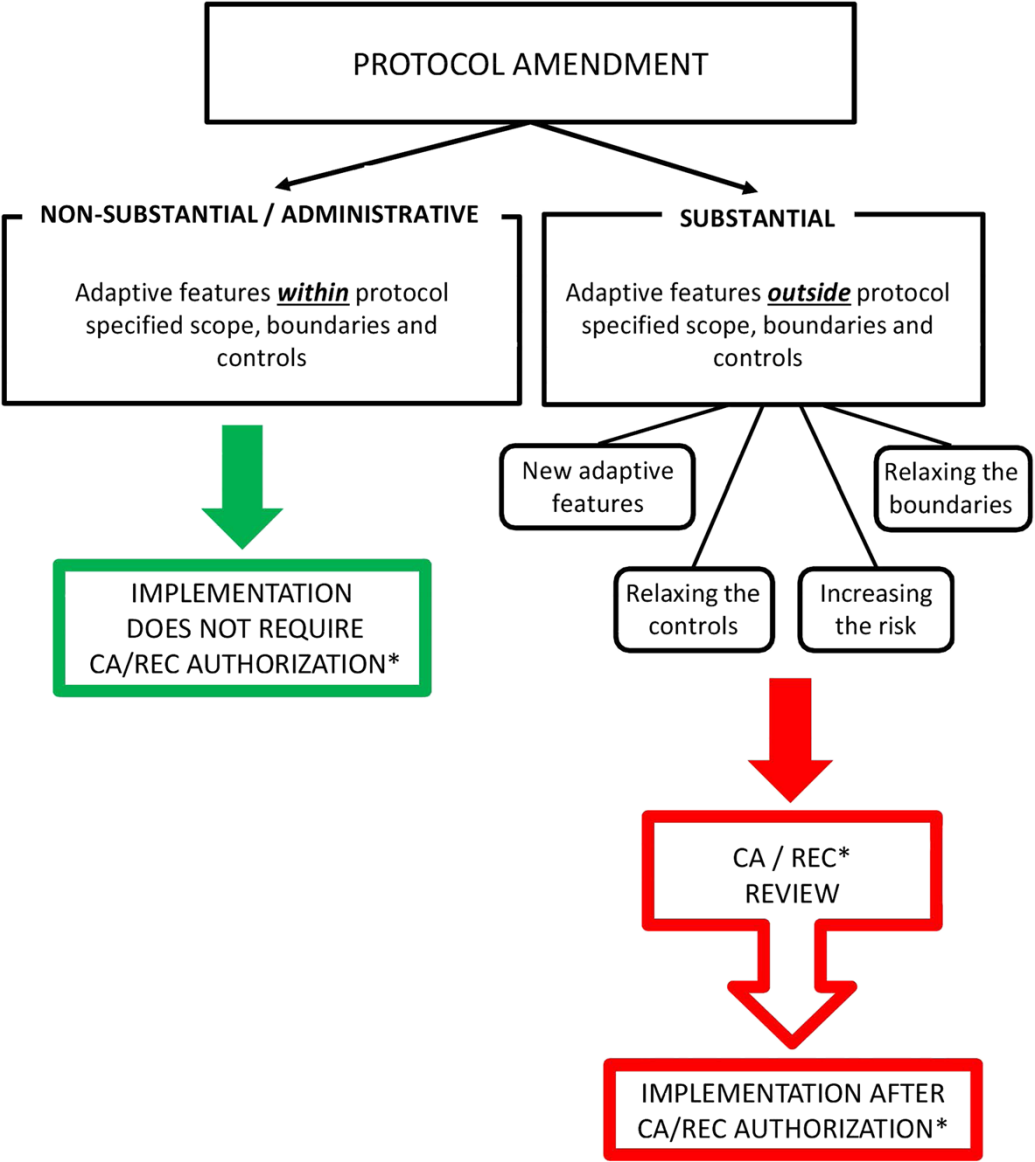
**Amendments for adaptive protocols.** *Medicines and Healthcare products Regulatory Agency (MHRA)/National Research Ethics Service (NRES), UK.

Changes *outside* the pre-defined scope of an adaptive protocol, its boundaries or control mechanisms constitute a substantial protocol amendment and require RA/REC approval as specified within legislation [6], Figure 1.

### Three steps to writing an early phase adaptive study protocol

#### Step 1: define and describe adaptive features

##### Terminology

*Adaptive features* are the characteristics of pre-defined adaptations that can be made to the protocol and study conduct.

##### Description

When defining adaptive features one needs to establish firstly which protocol areas will or may require flexibility to allow for adaptation, i.e. the *categories* of adaptations. Secondly, one needs to establish the details of potential adaptations, i.e. individual *adaptive features*. The use of some adaptive features will be certain from the outset (such as dose selection in a study where doses have not been set in the protocol), others will be optional (such as inclusion of more or less study participants, data analysis etc.). The categories and nature of adaptive changes that may potentially be required due to evolving data are largely predictable. Therefore, in an early phase protocol it is advantageous to make a full range of these potential adaptations available of which all necessary ones can be implemented without delay.

#### Step 2: define and describe boundaries

##### Terminology

*Boundaries* are limits that are agreed by the CA and describe the perimeter which potential adaptations are confined to, focussing on participants’ safety.

##### Description

Boundaries determine adaptive features’ maximum acceptable risk and inconvenience at the one end of the spectrum and minimum safety requirements at the other. Boundaries are set for each category and each of its individual adaptive features. Boundaries are an essential part of the risk management of a study. Regulatory acceptability of an adaptive trial depends on the setting of safe boundaries rather than the permutations and details of potential adaptations to the study conduct.

##### Template and examples for step 1 and step 2: adaptive features and their boundaries

Adaptive features and their boundaries can be systematically pre-defined and are best described in tabular format which makes all potential adaptations and their limits visible in one place. Tables 1, 2, 3, 4 and 5 provide a template with examples of adaptive features and their boundaries used in early phase studies authorised and performed in the UK.

**Table 1:** Investigational medicinal product/dose

**Adaptive study design category**	**Adaptive features**	**Boundaries**
**Dosing regimen**	1. Dosing regimens may be determined or adapted in accordance with pharmacokinetic (PK), pharmacodynamic (PD), safety and tolerability data (as applicable) collected up to the decision making time-point.	1. Maximum starting dose
	The term dosing regimen includes (1) the dose level administered, (2) the frequency of dosing and (3) the duration of dosing, i.e. the number of doses administered. Accordingly these can be adjusted individually or in combination.	2. Maximum dose or exposure increment for each dose/exposure escalation step
	2. The number of dosing regimens investigated may be adjusted.	3. Maximum (mean) exposure
		4. Minimum/maximum dosing frequency
		5. Minimum/maximum treatment duration
		6. Permissibility of dosing regimen adaptation within and/or between cohorts
		7. Minimum/maximum number of dosing regimens to be investigated, safety and tolerability permitting
		8. Dose/exposure relationships between discrete parts of umbrella protocols (e.g. between SAD and MAD parts).
**Sentinel/sub groups**	3. The number and size of sentinel/sub-groups groups within a dosing regimen may be adaptable	1. Mandatory sentinel groups for selected dosing regimens/study parts
		2. Maximum sentinel/sub-group group size; maximum number of study participants receiving IMP at any one time
		3. Minimum time to elapse between sentinel/sub-groups of a dosing regimen
**IMP formulation/mode of administration**	4. Adaptable use of different IMP formulations or modes of administration	1. Formulation/administration characteristics and requirements
		2. Exposure limits (see adaptive feature 1)

**Table 2:** Timing/Scheduling

**Adaptive study design category**	**Adaptive features**	**Boundaries**
**Overlap**	Dosing regimens or discrete parts of umbrella protocols may overlap	1. Minimum data requirements for progression between dosing regimens/protocol parts
		2. Reference to study specific toxicity rules

**Table 3:** Study participants

**Adaptive study design category**	**Adaptive features**	**Boundaries**
**Sample size**	1. The number of subjects in a dosing regimen/cohort can be decreased or increased.	1. Minimum data requirements for progression between dosing regimens/protocol parts
		2. Minimum/maximum size of a cohort/dosing regimen
		3. IMP/placebo ratio
		4. Reference to study specific toxicity rules
**Sample size**	2. The number of dosing regimens/cohorts may be decreased or increased	1. Minimum/maximum number of dosing regimens/cohorts (for each study part of an umbrella study), safety and tolerability permitting
		2. Reference to study specific toxicity rules
**Selection criteria**	3. Selection criteria may be adaptable	1. Defined criteria for which adaptability is permitted
		2. Criteria-specific direction and extent of adaptability

**Table 4:** Assessments

**Adaptive study design category**	**Adaptive features**	**Boundaries**
**Assessments**	1. Safety/tolerability samples and assessments (such as safety laboratory, vital signs, electrocardiograms (ECGs), continuous cardiac monitoring etc.) &	1. Minimum data requirements for progression between dosing regimens/protocol parts
	2. PK/PD/exploratory/other samples and assessments:	2. Defined sampling or assessment types/categories for which adaptability is permitted
	a) Additional or less samples or assessments may be taken	3. Minimum samples/time-points/assessments to sufficiently cover the full safety/tolerability and PK/PD profile in relation to relevant doses (e.g. single dose, steady state)
	b) Timing of samples or assessments may be adapted	4. Maximum
		-blood (or other) volumes
		-number of samples or assessments
		-sampling or assessment time-points
**Assessments**	3. Prolongation or shortening of the in-house period or out-patient follow-up period	1. Minimum/maximum in-house stay or out-patient follow-up periods based on:
		-study specific safety & tolerability, PK and/or PD parameters that must be reached prior to discharge from the clinic/study
		-evolving safety and tolerability profile of the IMP
		-and evolving PK/PD characteristics of the IMP up to the decision making time-point

**Table 5:** Methods and analysis

**Adaptive study design category**	**Adaptive features**	**Boundaries**
**Methods**	1. Methodology (such as food, cardiac safety and other clinical assessments, study specific techniques) may be adjusted	1. Defined methodologies for which adaptability is permitted
		2. Permitted purpose of adjustments
		3. Decision making time-points when adjustments may be made
**Analysis**	2. Optional analysis of safety, PK/metabolites/PD/exploratory/other samples/assessments:	1. Minimum data requirements for progression between dosing regimens/protocol parts
	a) Analysis may be limited to selected parameters, dosing regimens and time-points.	2. Defined sampling or assessment types/categories for which optional analysis is permitted
	b) Timing of optional analysis of certain parameters may be flexible	3. Permitted purpose(s) of optional analyses
		4. Reporting (how, where and when) of the optional analyses

In early phase clinical trials five overarching categories of adaptive features usually suffice: Investigational Medicinal Product (IMP)/Dose (Table 1), Timing/Scheduling (Table 2), Study Participants (Table 3), Assessments (Table 4), Methods and Analysis (Table 5). They are then broken down in further sub-categories (see Tables 1, 2, 3, 4 and 5; Column 1). Column 2 lists individual *adaptive features* within each of these four categories and their sub-categories. Column 3 lists the *boundaries* for each category and its adaptive features, wherever applicable.

Within the category of *assessments* (Table 4), due to lack of human data at the time of protocol writing, it may not be possible to set fixed boundaries for certain adaptive features. For instance, the schedule of assessments for First-in-Human studies will be largely based on pre-clinical data. The actual properties of the IMP in humans may prove to be different. Permissible assessment boundaries may therefore be difficult to determine at protocol writing stage. If that is so, rather than using arbitrary boundaries which later prove unsuitable, the protocol can include more general wording to describe principles and a process for their application, stipulating that adaptations should be made:

– in accordance with evolving data and dosing regimen up to the decision making time point;

– in the spirit of the current study protocol (i.e. focus on the capture of essential and useful data) not affecting the authorised risk profile of the study.

The UK competent authority (MHRA) is open to proposals for adaptations and will assess these on a case-by-case basis, taken in the wider context of the clinical trial.

#### Step 3: control mechanisms

##### Terminology

*Control mechanisms*: The mechanisms decision makers use to review data, to make and document decisions and to control progress of a study, namely *Study Progression Rules and Toxicity Rules.*

##### Description

During early phase adaptive studies, decision makers review evolving data at pre-defined decision making time-points using a defined process. The data is usually reviewed in a blinded fashion. Following review, decisions are made on study progression in accordance with the study’s options, i.e. its design, adaptive features and boundaries. The review meetings are minuted, the outcomes are documented. These documents become part of the Trial Master File.

##### Study progression rules

The elements of study progression rules which should be incorporated in an adaptive study protocol are:

(1) Decision making time-points

(2) Decision making process

 (a) Review team/decision makers

 (b) Blinded/unblinded review

 (c) Documentation of decision

(3) Minimum data reviewed at each decision making time-point

**Figure 2:**
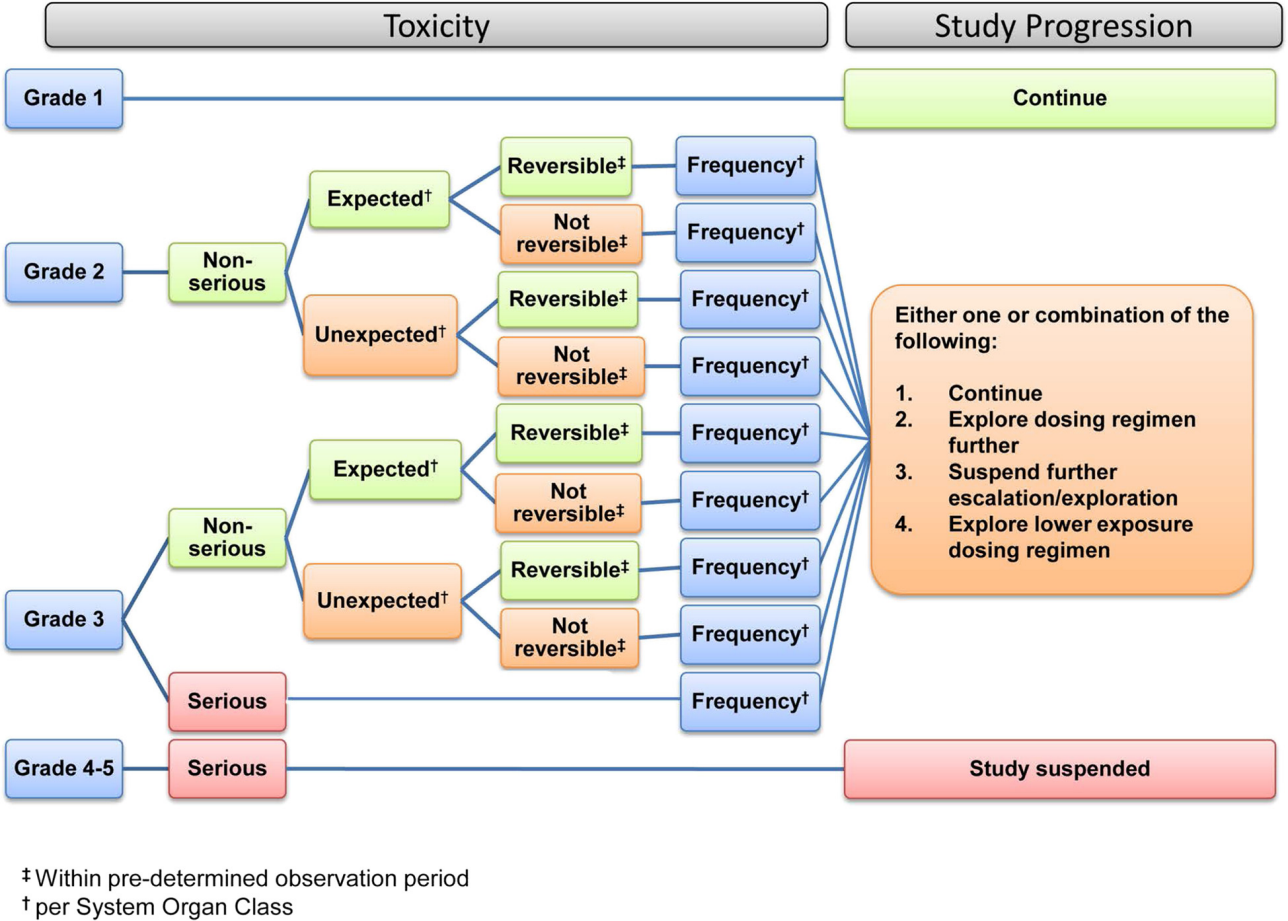
Toxicity rules.

 (a) Nature of the data (PK, PD, safety and tolerability (reviewed in accordance with toxicity algorithm, see Figure 2)

 (b) Number of subjects

 (c) Post-dose review time period

(4) Dependencies/next steps following data review at each decision making time-point

 a) Steps to proceed to distinct parts within an umbrella study

 b) Exposure/dose escalation steps within (parts of) a study

The detailed content of these protocol elements depend on the study design, the IMP PK/PD profile and its anticipated risks.

##### Template algorithm for step 3: study progression rules

**Figure 3:**
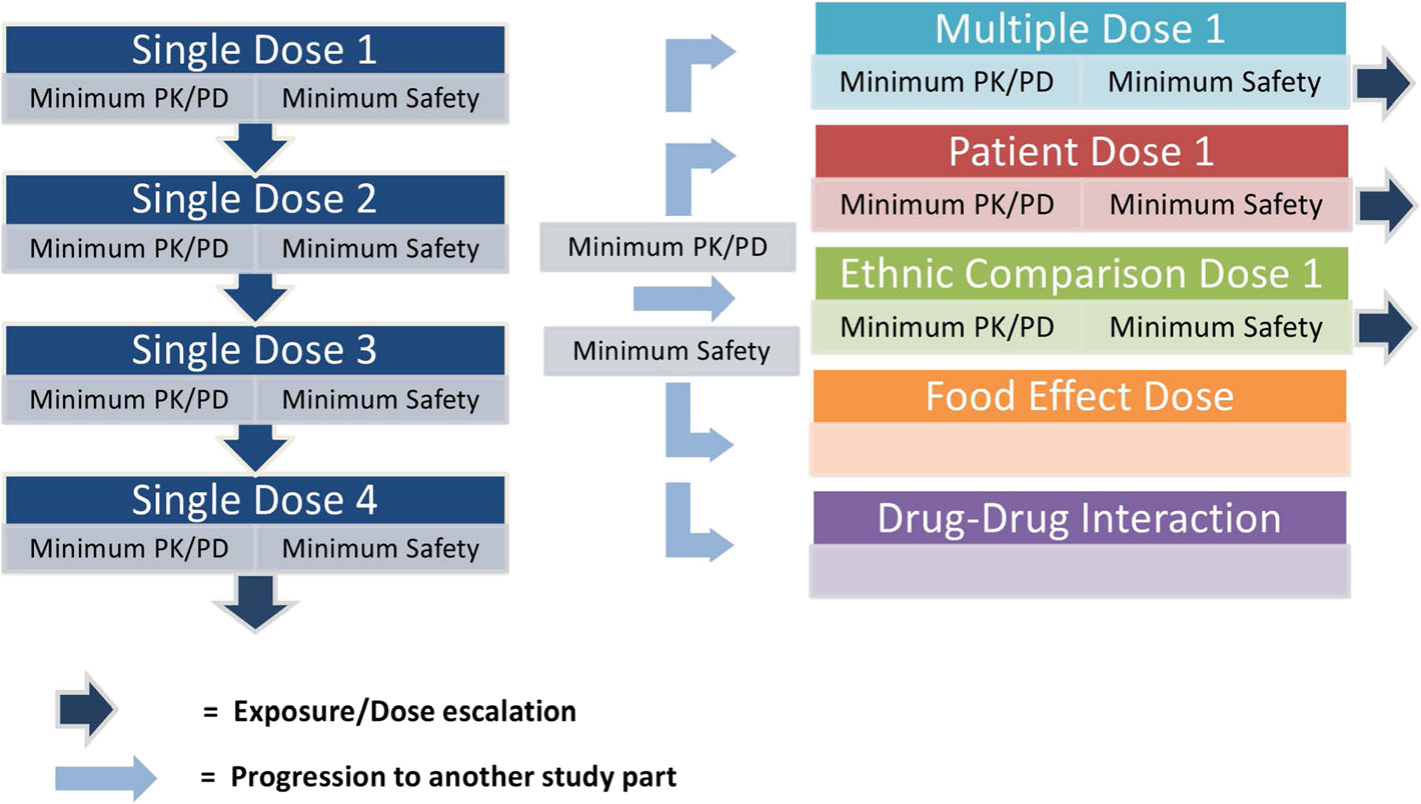
Study progression rules for an adaptive umbrella study.

The algorithm (Figure 3) visualises the decision making time-points, the minimum data reviewed at each decision making time-point and the next step(s) dependent on the data reviewed.

##### Toxicity rules

Toxicity rules can be efficiently described using standard terminology and template algorithms, adapted for each specific study. A suitable system for toxicity grading needs to be chosen, taking into consideration the nature of adverse reactions that may occur. For the purpose of this manuscript this includes adverse reactions that are *expected* in the regulatory sense, i.e. adverse reactions included in the Reference Safety Information (RSI) - with information on frequency and nature of the adverse reaction - for assessing whether a Serious Adverse Event (SAE) is classified as a Suspected Unexpected Serious Adverse Reaction (SUSAR).

There is usually no RSI during the first year of clinical development of new medicines, unless the RSI contained in the Investigator’s Brochure is updated via substantial amendments in the first year [6-8]. During this time, the “expectedness” of potential adverse reactions will be based on pre-clinical data and known class effects. This does not fall within the regulatory RSI definition but will nevertheless be clinically relevant for the development of study specific toxicity rules. Therefore the definition and basis of the term “expected” and the nature and frequency of “expected” adverse reactions need to be clearly described in the Investigator’s Brochure (e.g. in the Guidance for Investigators) and referenced in the study protocol.

The “Common Terminology Criteria for Adverse Events (CTCAE)” [9] provides terminology and toxicity grading for a wide range of adverse events. It was developed for oncology trials but can be used with the lower grading in early phase healthy volunteer and patient studies. The CTCAE is the most comprehensive reference document and based on “Medical Dictionary for Regulatory Activities” (MedDRA) terminology. There are other, more specific grading systems, such as the FDA’s toxicity grading for vaccine trials [10]. The chosen grading system should include suitable terminology for all “expected” adverse reactions. The CTCAE criteria and their interpretation are consistent with the standard intensity grading for Adverse Events during clinical studies: Grade 1 - mild, Grade 2 - moderate, Grade 3 - severe or medically significant, but not immediately life-threatening, may or may not constitute SAE/SUSAR. Grades 4 and 5 always constitute SAE/SUSAR.

Once a system for toxicity grading has been chosen, a *toxicity rules* algorithm is developed for the proposed study (Figure 2), taking into account toxicity grading, severity/seriousness, reversibility, “expectedness” and frequency. Based on these input factors, the algorithm leads to study specific actions and effects on study progression, minimising risk.

##### Template algorithm for step 3: toxicity rules

The frequency of Grade 1 toxicities has often little impact on study progression in early phase studies. Reversibility within a pre-determined observation period and “expectedness” are factors that are usually most relevant in the consideration of Grade 2 and non-serious Grade 3 toxicities, when decisions on study progression are being made. There may be compounds for which this is different, in which case the template algorithm needs adjusting. The occurrence of one case of a serious Grade 3 toxicity would normally suspend further dosing at this exposure level and further dose escalation. Study continuation at a lower exposure level may be permissible. The occurrence of Grade 4 or Grade 5 toxicity in a single study participant would normally suspend a study.

Maintaining the blinding whilst applying the toxicity algorithm is not problematic, unless higher grade, potentially drug related toxicities occur which may lead to suspension of the study. In such cases, decision makers may decide to have the relevant data reviewed unblinded. If appropriate, this can be done in the first instance by an independent party, maintaining the investigational staffs’ and decision makers’ blinding.

### Practical considerations

#### Protocol development

A common objection raised in relation to adaptive studies is the seemingly complex design and protocol development. The potential introduction of bias undermining the validity and integrity of the study is another concern commonly raised. Regulatory acceptability of any type of protocol depends on a clear description and justification of a study’s design and its risk management. Study endpoints and the management of potential risks are the main factors considered when setting adaptive features, boundaries and control mechanisms. This is however not specific to adaptive study design; these factors need to be considered for any type of protocol, whether adaptive or non-adaptive.

This manuscript shows how the use of a systematic, standardised 3-step approach can assist the efficient writing of a complete adaptive protocol. Templates can be adapted to specific studies and used as checklists to ensure all potential adaptive features, their boundaries and study control mechanisms have been considered and fully described. Provided that such a standard template is used and operational and technical detail is described in an operational manual, the writing of an adaptive protocol is no more complex than the writing of a well-considered, non-adaptive protocol. In fact, the writing of an adaptive protocol may be less challenging than the writing of a non-adaptive protocol; the latter requires accurate predictions of all potential outcomes. Moreover, all predictions must subsequently be found to be correct in order to enable completion in accordance with the original study protocol. Failing that, ad-hoc substantial protocol amendments must be made and approved prior to continuing a non-adaptive study. Conversely, an adaptive protocol allows well considered and pre-defined adaptations within their pre-specified boundaries. Adaptive protocols avoid ad-hoc changes to a study protocol and the resulting potential introduction of bias. An adaptive study can continue to proceed in accordance with the original protocol.

### Implementation of adaptive changes

The flexibility and time savings [11] of an adaptive design may be lost if interim data at decision making time points and proposed adaptive changes need to be disseminated to or authorised by the CA or REC. The UK has a favourable environment for the conduct of adaptive studies. The approval of the study protocol is based on the agreed parameters in terms of acceptable risk and participant inconvenience, ring-fenced by the adaptive scope, boundaries and control mechanisms, with a clear focus on participants’ safety. Once a study protocol has been approved, there is no further interaction with the CA/REC so long as the study proceeds within the protocol’s pre-defined adaptive specifications. Interactions with CA/REC are only required if major changes to the protocol are proposed, i.e. substantial amendments outside its adaptive specifications, such as for example increasing the pre-defined maximum exposure limit, because this could change the approved balance between risk and benefit.

It is not the role of the CA or REC to routinely check compliance with the protocol and its approved decision making processes whilst a study is ongoing. This aspect is dealt with by distinct Quality Assurance processes such as audits, inspections and in the UK also the MHRA Phase 1 Accreditation scheme [12]. Any significant safety signals will become known to the CA/REC in any case, as they would either lead to suspension of a study or a substantial protocol and/or RSI amendment.

### Safety

A question raised in relation to adaptive protocol design is whether it may increase the risk for study participants. We believe that adaptive studies can be inherently safer than non-adaptive studies. Adaptive protocols require *by design* a continuous assessment of evolving data and well documented risk management processes. If the protocol is written as we propose in this manuscript, the maximum acceptable risk and inconvenience to participants are clearly confined within a protocol’s adaptive specifications. Adaptive features remove obstacles to making changes mandated by new safety data. Finally, adaptive design avoids collection of unnecessary data and unnecessary exposure to participants.

## Summary

Adaptive protocol design has universal use across early phase clinical research. The adaptive concept of using evolving data to modify the trial design during clinical trial conduct within the protocol-defined remit is efficient in gathering meaningful and relevant data, ethical and time- and cost-effective.

The simple 3-step process of adaptive protocol writing described in this manuscript may support the wider use of adaptive protocol design in exploratory early phase clinical research.

## Abbreviations

CA: Competent authority; CTCAE: Common terminology criteria for adverse events; EMA: The European Medicines Agency; FDA: U.S. Food and Drug Administration; IMP: Investigational medicinal product; MAD: Multiple ascending dose; MedDRA: Medical dictionary for regulatory activities; PD: Pharmacodynamics; PK: Pharmacokinetics; RA: Regulatory authority; REC: Research ethics committee; RSI: Reference safety information; SAD: Single ascending dose; SAE: Serious adverse event; SUSAR: Suspected unexpected serious adverse reaction.

## Competing interests

The authors declare that they have no financial competing interests.

MO declares that the views presented in this publication are those of the author and should not be understood or quoted as being made on behalf of the MHRA and/or its scientific committees. Views are presented solely to aid the discussion and should not be interpreted as adopted guidance.

## Authors’ contributions

UL prepared the current manuscript. MO provided a regulatory review. JT supervised the process of writing and revised the manuscript critically for important intellectual content. All authors read and approved the final manuscript.

## Pre-publication history

The pre-publication history for this paper can be accessed here:

http://www.biomedcentral.com/1471-2288/14/84/prepub
